# PathoBacTyper: A Web Server for Pathogenic Bacteria Identification and Molecular Genotyping

**DOI:** 10.3389/fmicb.2017.01474

**Published:** 2017-08-03

**Authors:** Ming-Hsin Tsai, Yen-Yi Liu, Von-Wun Soo

**Affiliations:** ^1^Institute of Population Health Sciences, National Health Research Institutes Miaoli County, Taiwan; ^2^Department of Computer Science, National Tsing Hua University Hsinchu, Taiwan

**Keywords:** next-generation sequencing, pan-genome database, whole genome multilocus sequence typing, molecular typing, bacterial identification

## Abstract

With the decline in the cost of whole-genome sequencing because of the introduction of next-generation sequencing (NGS) techniques, many public health and clinical laboratories have started to use bacterial whole genomes for epidemiological surveillance and clinical investigation. For epidemiological and clinical purposes in this “NGS era,” whole-genome-scale single nucleotide polymorphism (wgSNP) analysis for genotyping is considered suitable. In this paper, we present an online service, PathoBacTyper (http://halst.nhri.org.tw/PathoBacTyper/), for pathogenic bacteria identification and genotyping based on wgSNP analysis. More than 400 pathogenic bacteria can be identified and genotyped through this service. Four data sets containing 59 *Salmonella* Heidelberg isolates from three outbreaks with the same pulsed-field gel electrophoresis pattern, 34 *Salmonella* Typhimurium isolates from six outbreaks, 103 isolates of hospital-associated vancomycin-resistant *Enterococcus faecium* and 15 *Legionella pneumophila* isolates from clinical and environmental samples in Israel were used for demonstrating the operation and testing the performance of the PathoBacTyper service. The test results reveal the applicability of this service for epidemiological typing and clinical investigation.

## Introduction

The genomic DNA of organisms carries genetic information that is biologically functional. Decoding the entire genome sequence of an organism is a fundamental task in complex biological studies. Previously, the conventional Sanger sequencing method was used to decode the complete bacterial genome sequence; however, this method is very expensive and tedious. In recent years, considerable progress has been made in next-generation sequencing (NGS) technology. Currently, the NGS method can facilitate bacterial genome decoding within days and at a cost of less than US$100. Therefore, in the near future, whole-genome sequencing (WGS) is expected to be used in clinical and public health laboratories and to become a routine diagnostic and genotyping tool for disease surveillance, resistance prediction, cluster infection examination, and establishing evolutionary relationships among different strains (Parkhill et al., 2001; Merker et al., 2013; Struelens and Brisse, 2013; Franz et al., 2014; Gordon et al., 2014; Halachev et al., 2014; Joensen et al., 2014; Koser et al., 2014; Luo et al., 2014; Schmid et al., 2014). In addition to its clinical and public health applications, WGS is a very effective method for basic biomedical research such as studies on the pathogenesis of human diseases (Acke et al., 2014; Hoffmann et al., 2014; Meinel et al., 2014). However, the NGS platform typically generates millions of short sequences, and the analysis of such a large number of short WGS sequences to generate the required information, such as the genotype and resistance to different strains, is a challenge. Because most researchers in clinical and public health laboratories lack expertise in bioinformatics, developing a simple and easy-to-use analytical platform for automating the analysis of WGS primitive sequence fragments and for performing genotypic comparison of different strains in the laboratory is necessary.

The whole-genome-scale single nucleotide polymorphism (wgSNP) approach has been demonstrated to be suitable for bacterial strain genotyping (Achtman, 2008; Nielsen et al., 2011; Leekitcharoenphon et al., 2014). Many researchers have successfully applied wgSNP analysis for detecting outbreaks of different types of pathogenic bacteria (Holt et al., 2010; Bakker et al., 2011; Octavia et al., 2015; Taylor et al., 2015; Bekal et al., 2016). Several effective tools such as Lyve-SET (Katz et al., 2017), SNVPhyl (Petkau et al., 2016), and CFSAN SNP Pipeline (Davis et al., 2015) have been designed for manipulating WGS data to generate wgSNP metadata. However, these wgSNP tools are usually deployed as command-line programs, which is inconvenient and difficult for most wet-lab employees who must manipulate the WGS data in clinical and public health laboratories. Therefore, first-line lab personnel have a high demand for an easy-to-use tool that can help them generate wgSNP metadata from WGS data for further applications.

In this paper, we present a web-service tool, PathoBacTyper, for pathogenic bacterial identification and molecular genotyping of more than 400 pathogenic bacteria. In this study, we demonstrated the operation of PathoBacTyper by identifying and genotyping four data sets, such as 59 *Salmonella* Heidelberg WGS raw reads from three outbreaks previously sequenced by Bekal et al. (2016), 34 *Salmonella* Typhimurium isolates (Leekitcharoenphon et al., 2014), 103 isolates of hospital-associated vancomycin-resistant *Enterococcus faecium* (De Been et al., 2015) and 15 *Legionella pneumophila* isolates from clinical and environmental samples in Israel (Moran-Gilad et al., 2015). The 34 *Salmonella* Typhimurium isolates are consisted of 18 strains from six previously studied outbreaks (Torpdahl et al., 2007; Petersen et al., 2011) and 16 unrelated strains. The 103 *E. faecium* strains are comprised of 46 strains isolated from German hospital during 2003 to 2006, 37 isolates from Danish hospitals during 2012–2013, and 20 isolates from Dutch hospitals during 2012 to 2013. The test results reveal the applicability of this service for epidemiological typing and clinical investigation.

## Methods and Implementation

**FIGURE 1:**
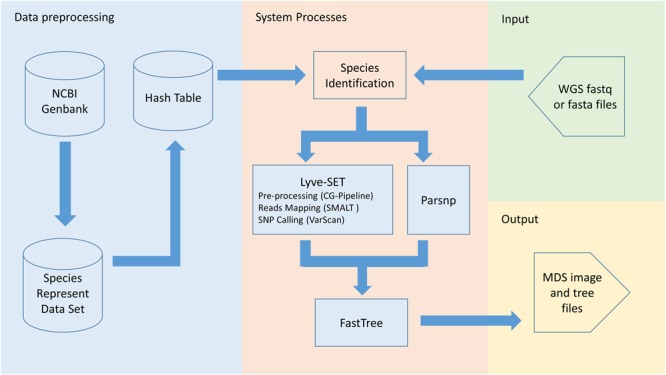
The schematic work flow of PathoBacTyper.

PathoBacTyper provides two functions: species identification and bacterial strain typing. In species identification, user-uploaded WGS reads are distributed to a prebuilt reference data set comprising 478 pathogenic bacterial genomes; possible species can then be identified according to the quantity of the mapped reads. After the species of the user-uploaded isolate whole genome sequence is recognized, the corresponding reference sequence of the species is automatically selected for the following strain typing process. **Figure 1** presents the overall workflow of PathoBacTyper. A detailed description of the methodologies used in PathoBacTyper for species identification and bacterial strain typing follows.

### Species Identification

In the species identification process, the user-uploaded bacterial isolate WGS reads are mapped to a species genome reference database (SGRdb) comprising 478 pathogenic bacteria, which were selected from the list provided by Scholz et al. (2016). The isolates included in the SGRdb are presented in Supplementary Table S1. For mapping the reads, we applied the hash-based method (Alkan et al., 2009; Weese et al., 2009), which transforms tens of thousands of read sequences into a k-mer patterned hash table, and then the best match is rapidly obtained through comparison with the hash tables created from the reference genomes (i.e., genomes from the SGRdb in our case). In this study, a 30-mer hash table for the SGRdb (SGRdbht) was constructed using a sliding window with a 30-mer step size. The system creates hash keys by using the uploaded raw reads to query the SGRdbht for the species identification process. A coverage ratio is defined to evaluate the support level of the candidate sequence from the SGRdb. The coverage ratio is defined as *C* = *R*/*M*, where *C* is the coverage ratio, *R* denotes a number of mapping raw reads hash keys onto the positions of candidate sequence, and *M* denotes a total number of hash positions on the candidate sequence.

### Bacterial Strain Typing

In the bacterial strain typing process, the user-uploaded WGS raw reads and the reference genome, which is selected from the SGRdb on the basis of the identification results from the species identification process or is uploaded by the user, are used as the input. We selected the Lyve-SET (Katz et al., 2017) method for calling wgSNPs; this method creates high-quality wgSNPs from WGS raw reads. The distance matrix computed using Lyve-SET is used for depicting a multidimensional scaling (MDS) plot, which can provide a good presentation of outbreak clusters. If input files are assembly contigs, WgSim is used to simulate raw reads in Lyve-SET. To omit the extra reads simulation process, the Parsnp (Treangen et al., 2014) method replaces Lyve-SET when assembly contig files are uploaded. In the process of calculating SNPs with assemblies, Parsnp is more accurate and faster than Lyve-SET. The FastTree (Price et al., 2010) is used to calculate the alignment files, which are outputted from both Lyve-SET and Parsnp to build phylogenetic trees. Moreover, we constructed a maximum-likelihood phylogenetic tree with confidence values labeled on the branches by using the ETE toolkit (Huerta-Cepas et al., 2016).

### Implementation

The PathoBacTyper service was built by integrating the species identification and bacterial strain typing functional modules in Java programs. The web page was constructed using HTML, JavaScript, and JSP. The service runs on a Linux server with two 2.40-GHz Intel Xeon processors comprising 16 cores.

## Web Server

### Input Format

**FIGURE 2:**
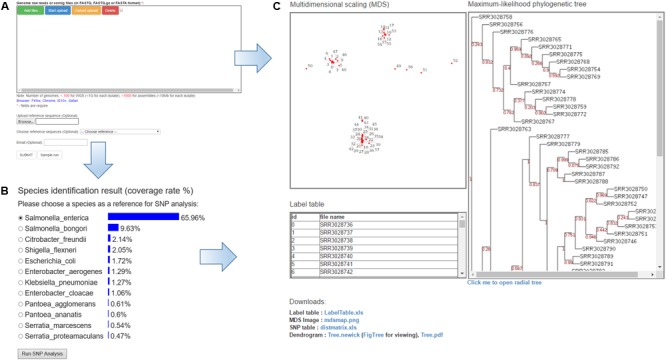
The features of the PathoBacTyper server. **(A)** Input page. **(B)** Result page of the *Species identification*. **(C)** Output page of the results, such as phylogenetic tree MDS and label table.

PathoBacTyper accepts two genomic sequence formats, “fastq” and “fasta.” The fastq data can be in the gzip format (.gz). All upload files are deleted after the analysis is completed, and the size of every uploaded file cannot exceed 1 GB. PathoBacTyper typically takes 587 and 101 min on average to finish all the processes for a data set comprising 59 *S*. Heidelberg genomes in the fastq and fasta formats, respectively. Users are encouraged to provide e-mail addresses for obtaining notification of the results when their jobs are finished. The home page for the user to upload WGS data is shown in **Figure 2A**.

### Output Format

The PathoBacTyper output is composed of two sections, species identification (**Figure 2B**) and SNP analysis (**Figure 2C**). In the species identification part, the system lists the top 10 candidate species, ranked by confidence values. For the SNP analysis part, the result page includes (A) an MDS image file; (B) a label table, which provides a link from MDS labels to the upload file name; (C) a maximum-likelihood phylogenetic tree (in the pdf and png image file formats); and (D) a phylogenetic tree text file in the Newick format. The Newick tree file can be reused to draw the phylogenetic tree in other software. If users enter their email addresses on the job submission page, the system sends them a link to the URL of the result page when the job is finished.

## Example Analysis

Four data sets are used to evaluate our implementation, such as 59 *S.* Heidelberg isolate genomes from three outbreaks with an identical pulsed-field gel electrophoresis type (Bekal et al., 2016), 34 *S.* Typhimurium isolate genomes from six outbreaks (Leekitcharoenphon et al., 2014), 103 isolates of hospital-associated vancomycin-resistant *E. faecium* (De Been et al., 2015), and 15 *L. pneumophila* isolates (Moran-Gilad et al., 2015). The strain name and ENA accession number of 103 *E. faecium* isolates and 15 *L. pneumophila* isolates are listed in the Supplementary Table S4, S5 separately. In *E. faecium* analysis, The PathoBacTyper successfully identified each subtype, as shown in Supplementary Figure S1. The Supplementary Figure S2 shows the result of *L. Pneumophila* analysis. The strains labeled by Lp-001, Lp-2002694p7, and Lp-012 are far from the major cluster, that is similar to the spanning tree in the original paper. Other strains are clustered that is consistent with literature data. Other details are demonstrated in following sections.

### Demonstration of the Species Identification and the Molecular Typing Functionality for *S*. Heidelberg

**FIGURE 3:**
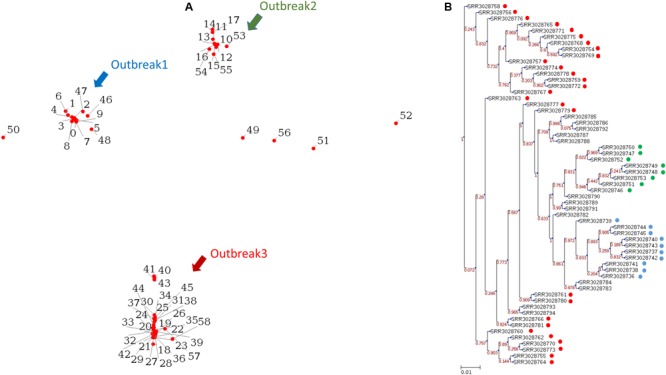
Reveals the 59 *S.* Heidelberg isolates from three outbreaks, which are identified as distinct clusters in both the MDS plot **(A)** and phylogenetic tree **(B)**. The blue, green, and red circles represent the outbreaks outbreak 1, outbreak 2, and outbreak 3 separately in **(B)**.

We tested the operation of PathoBacTyper by using a data set comprising 59 *S*. Heidelberg isolate genomes from three outbreaks with an identical pulsed-field gel electrophoresis type (Bekal et al., 2016). The WGS raw reads of these 59*S*. Heidelberg isolates (Supplementary Table S2) were downloaded from the NCBI SRA database^1^. The SRA Toolkit^2^ was used to convert the downloaded raw reads in the sra format to the fastq format. Moreover, we *de novo* assembled all 59 *S*. Heidelberg genomes with CLC v9.5.2. For processing the example data, approximately 9.5 h were required on a Linux server with two 2.40-GHz Intel Xeon processors comprising 16 cores to finish all the processes for outputting the MDS plot and the phylogenetic tree. We spent 19 min and approximately 1 min, depending on network traffic, uploading all 59 *S*. Heidelberg genomes in the fastq (raw reads) and fasta (assemblies) formats, respectively. The process times of the test data set were 587 and 101 min for raw reads and assemblies, respectively. The results of raw reads reveal that three outbreaks can be identified as distinct clusters in both the MDS plot (**Figure 3A**) and phylogenetic tree (**Figure 3B**). As shown in **Figure 3B**, the isolates differed at 10 SNPs or less within the same outbreak and over 50 SNPs among distinct outbreaks. The genetic relationships among the 59 isolates were highly concordant with the epidemiological definitions in a previous study (Bekal et al., 2016). We compared the results between the two different inputs, raw reads and assemblies (Supplementary Figure S3). Although both tree topologies were not identical, the three outbreaks can be clearly distinguished.

### Demonstration of the Species Identification and the Molecular Typing Functionality for *S*. Typhimurium

**FIGURE 4:**
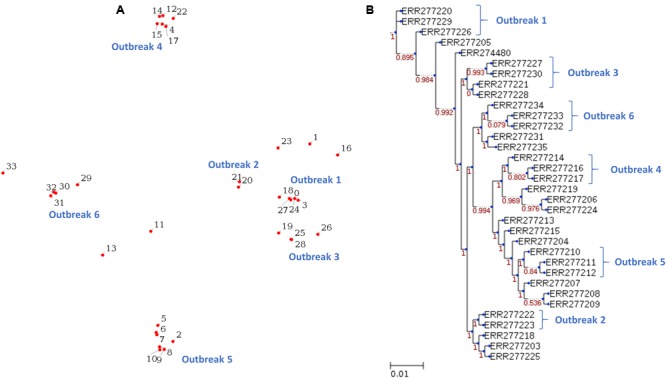
Reveals the 34 *S*. Typhimurium isolates from six outbreaks, which are identified as distinct clusters in both the MDS plot **(A)** and phylogenetic tree **(B)**. The six outbreaks are labeled with outbreak 1–6 in **(A, B)**.

The 34 isolates of *S*. Typhimurium are consisted of 18 strains from six outbreaks and 16 unrelated strains (Leekitcharoenphon et al., 2014). The WGS raw reads of these 34 *S*. Typhimurium isolates (Supplementary Table S3) were downloaded from the NCBI SRA database. Same as above section, the raw reads are converted to fastq format by the SRA Toolkit. The results illustrate that six outbreaks can be identified as distinct clusters in both the MDS plot (**Figure 4A**) and phylogenetic tree (**Figure 4B**). We tested the operation of PathoBacTyper with four data sets to confirm our implement is stable enough, and the output is consistent with literature.

## Discussion

The main purpose of our study is to implement a convenient platform for researchers who are interested in epidemiological typing and clinical investigation. The PathoBacTyper accepts two types of sequences, raw reads and assembly contigs, and no more parameters are required. This website provides a visualization result page, MDS plot and phylogenetic tree, which lets users easier to understand relationship among outbreaks. Furthermore, we provide several download links for users to validate the analysis results or redraw the tree in their favorite style. However, the computing power seems not sufficient to satisfy all requests from internet. The NGS sequences alignment essentially consume a lots of computing resource, therefore a standalone version of PathoBacTyper is needed. Additionally, the function of species identification is limited if user upload a species which is not included in the database SGRdb. To solve the problems mentioned above, we provide PathoBacTyper as a virtual machine image in the download page^3^. Users can download and run PathoBacTyper on their own server that allows new species addition and the SGAdb rebuilding. However, a virtual machine deployment and manual operation of species addition are inconvenient. Therefore, a standalone GUI-based tool, such as java application, with automatic new species addition and SGAdb rebuilding is needed and will be implemented in our next version of PathoBacTyper.

## Conclusion

The PathoBacTyper web server comprising two functions, species identification and bacterial strain typing, was established for researchers to perform epidemiological typing and clinical investigation for more than 400 pathogenic bacterial organisms. With NGS becoming a routine approach in clinical and public health laboratories, a research use only analysis platform for handling hundreds of thousands of WGS data is crucial. Such molecular genotyping will provide an information about strain-relatedness among outbreaks to support the infection control by field study. Through the PathoBacTyper service, users can directly upload WGS raw reads or assemblies of bacterial isolates for performing species identification and strain typing without expertise in bioinformatics. We believe that PathoBacTyper is a very powerful online tool for disease outbreak investigation and surveillance.

## Author Contributions

M-HT and Y-YL conceived and designed the experiments. M-HT and Y-YL performed the experiments and analyzed the data. M-HT and Y-YL contributed materials/analysis tools. M-HT, Y-YL, and V-WS wrote the paper.

## Conflict of Interest Statement

The authors declare that the research was conducted in the absence of any commercial or financial relationships that could be construed as a potential conflict of interest.

## Footnotes

1 https://www.ncbi.nlm.nih.gov/sra

2 https://github.com/ncbi/sra-tools

3 http://halst.nhri.org.tw/PathoBacTyper/download.jsp
