# Supplementary figures and images for: PathoBacTyper: A Web Server for Pathogenic Bacteria Identification and Molecular Genotyping
Source: Front Microbiol. 2017 Aug 3;8:1474. doi: 10.3389/fmicb.2017.01474 (PMC5540972; doi:10.3389/fmicb.2017.01474)

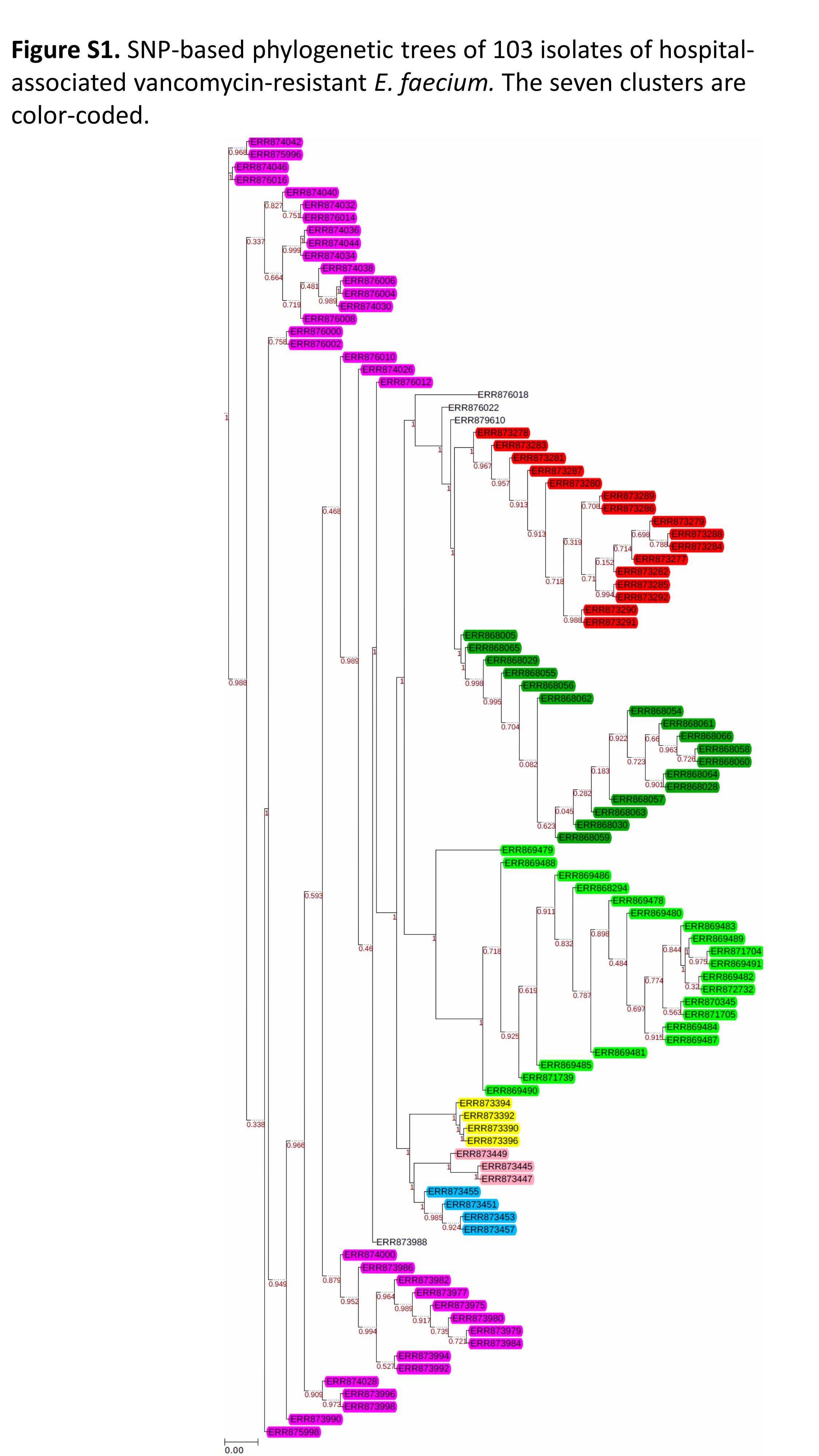

Supplement: Supplementary file 6 [file Image_1.TIF]

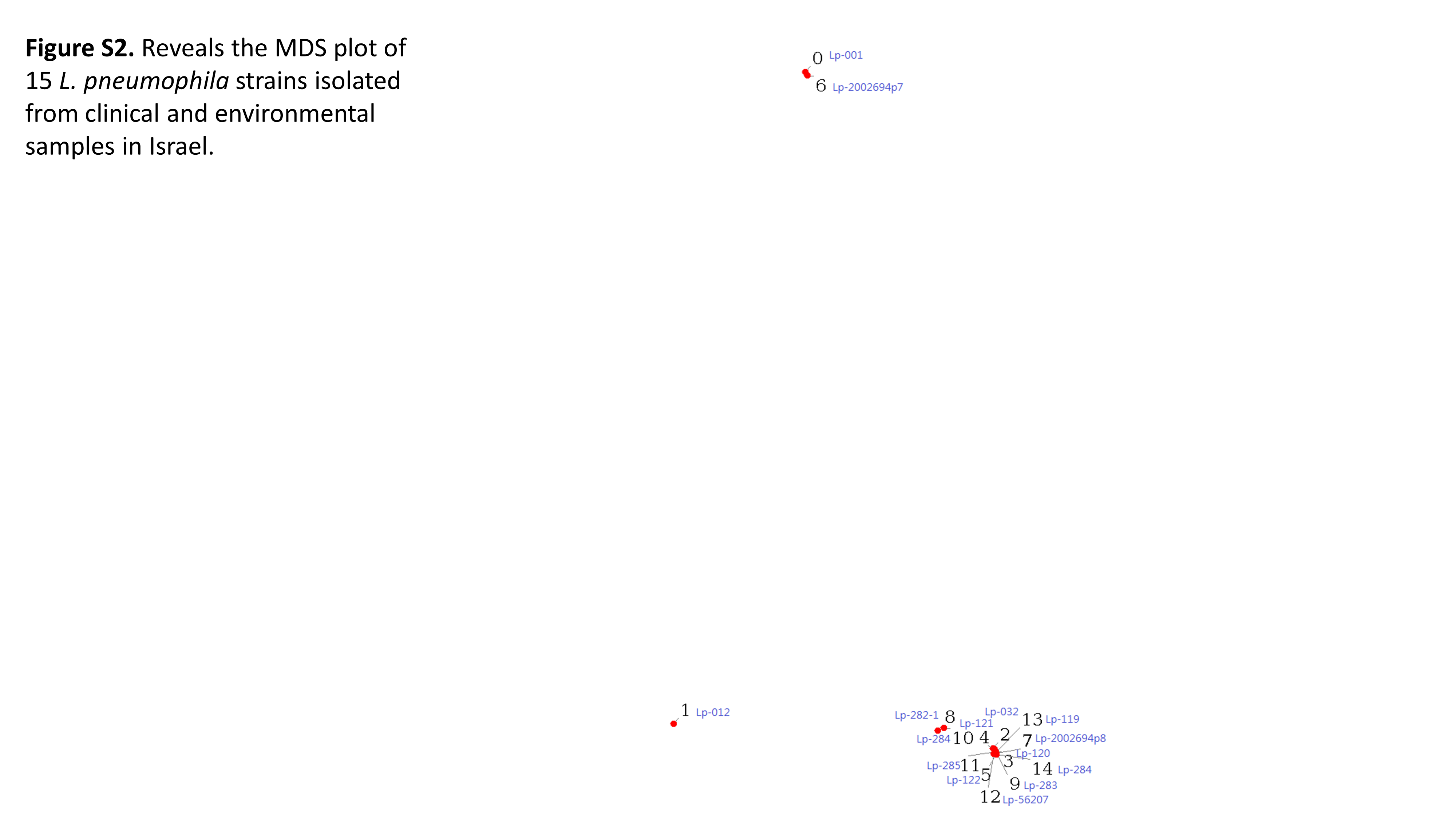

Supplement: Supplementary file 7 [file Image_2.TIF]

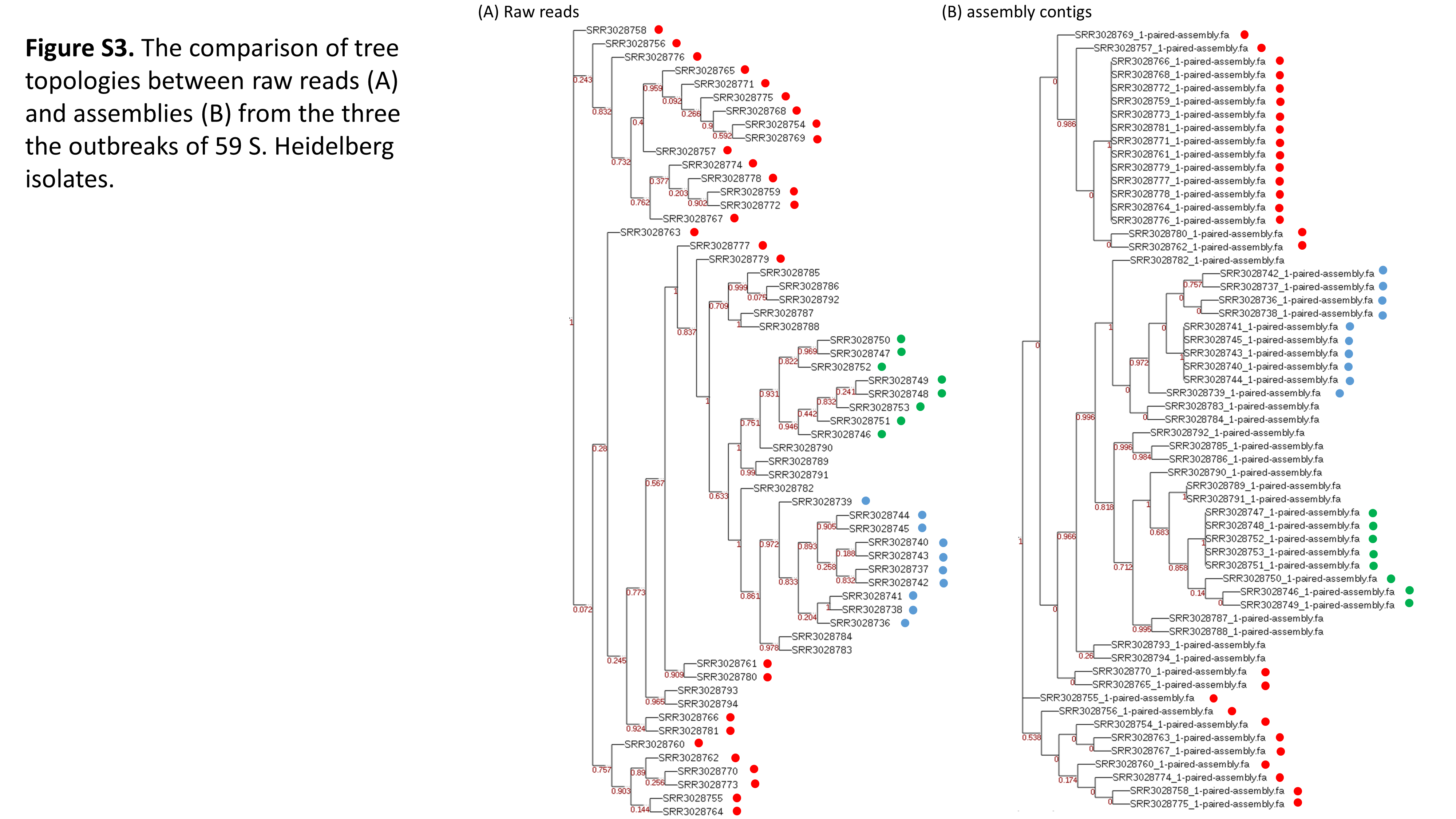

Supplement: Supplementary file 8 [file Image_3.TIF]
